# Role of Epidural Electrode Stimulation in Three Patients with Incomplete AIS D Spinal Cord Injury

**DOI:** 10.3390/biomedicines13010155

**Published:** 2025-01-10

**Authors:** Yu-Chen Chen, Xiang-Ling Huang, Hung-Yu Cheng, Ciou-Chan Wu, Ming-Yung Wu, Lian-Cing Yan, Shin-Yuan Chen, Sheng-Tzung Tsai, Shinn-Zong Lin

**Affiliations:** 1 Departments of Neurosurgery, Hualien Tzu Chi Hospital, Buddhist Tzu Chi Medical Foundation, No. 707, Sec. 3, Zhongyang Rd., Hualien City 970, Hualien County, Taiwan; spring810569@gmail.com (Y.-C.C.); shinnzong@yahoo.com.tw (S.-Z.L.); 2 Department of Medical Informatics, Tzu Chi University, No. 701, Sec. 3, Zhongyang Rd., Hualien City 970, Hualien County, Taiwan; 3 Department of Nursing, Hualien Tzu Chi Hospital, Buddhist Tzu Chi Medical Foundation, No. 707, Sec. 3, Zhongyang Rd., Hualien City 970, Hualien County, Taiwan; 4 Institute of Medical Sciences, Tzu Chi University, No. 701, Sec. 3, Zhongyang Rd., Hualien City 970, Hualien County, Taiwan; 5 Department of Physical Medicine and Rehabilitation, Hualien Tzu Chi Hospital, Buddhist Tzu Chi Medical Foundation, No. 707, Sec. 3, Zhongyang Rd., Hualien City 970, Hualien County, Taiwan; 6 School of Medicine, Tzu Chi University, No. 701, Sec. 3, Zhongyang Rd., Hualien City 970, Hualien County, Taiwan; 7 Department of Medicine, Tzu Chi University, No. 701, Sec. 3, Zhongyang Rd., Hualien City 970, Hualien County, Taiwan

**Keywords:** epidural electrical stimulation, spinal cord injury, walking function, walking symmetry

## Abstract

**Background/Objectives**: To determine whether epidural electrical stimulation (EES) improves sensory recovery and walking function in patients with chronic spinal cord injury (SCI) with a grade on the American Spinal Cord Injury Association impairment scale (AIS) of C or D at the cervical level. **Methods**: Three individuals with cervical-level chronic AIS D SCI were enrolled in the study. The mean injury duration and age were 4.8 ± 4.5 (range: 1.5–10) and 56.7 ± 9 years, respectively. The participants received personalized electrical stimulation for 36 weeks and were evaluated for their SCI characteristics, the result of an AIS assessment according to the lower extremity sensorimotor scale, their muscle activity, and preoperative walking ability parameters, initially as well as at weeks 8 and 36 of the EES intervention. **Results**: Participants receiving EES significantly increased the muscle activity in most lower limb muscles. Regarding the AIS assessment of the lower extremities, one participant fully regained a light touch sensation, while two fully recovered their pinprick sensation (AIS sensory scores increased from 14 to 28). One participant achieved a full motor score, whereas the others’ scores increased by 19 and 7 points. Compared with preoperative gait parameters, two participants showed improvements in their walking speed and cadence. Walking symmetry, an important parameter for assessing walking function, improved by 68.7%, 88%, and 77% in the three participants, significantly improving the symmetry index (*p* = 0.003). **Conclusions**: Thus, EES may be an effective strategy for sensory impairment recovery, as well as muscular activity and strength improvement. These findings may facilitate stable walking in subjects with chronic incomplete SCI, but larger clinical trials are warranted. **Clinical** trial: NCT05433064.

## 1. Introduction

Spinal cord injury (SCI) can have profound and enduring impacts on an individual’s life. The long-term effects largely depend on the injury’s location and severity [1]. Motor and sensory impairment is one of the most prevalent and debilitating consequences, often leading to the loss of voluntary muscle control and strength beneath the injury level. For instance, injuries to the cervical spine can result in tetraplegia, whereas in thoracic or lumbar regions, such injuries might cause paraplegia [2]. These impairments can significantly hamper mobility, challenging walking and standing, as well as hand function. Common symptoms include muscle weakness, sensory deficits, and spasticity.

Thus, physical therapy and rehabilitation are key to recovery [3]; despite intensive rehabilitation efforts, many patients with SCI in the chronic phase still experience limited motor improvement [4]. Recently, neuromodulation, particularly through epidural electrical stimulation (EES), has become a promising intervention for neurological disorders causing motor impairment [5,6]. EES involves electrode implantation coupled with a pulse generator to administer specific electrical impulses to the epidural space of the spinal cord and stimulate specific muscle contraction [7]. This stimulation can activate neural pathways below the injury site [8], with the potential to improve motor functions [9,10,11,12].

EES has been demonstrated to help individuals with SCI regain voluntary movements [8,13]; previous studies have shown that EES and rehabilitation facilitate the volitional control of motor activity, including trunk stability, independent standing, stepping, and even walking over the ground with assistive devices or body weight support, in participants with grades on the American Spinal Cord Injury Association impairment scale (AIS) of A and B [12,14,15,16,17,18,19,20,21,22,23,24]. Moreover, EES helps with regaining autonomous activity, ambulatory movements, and walking in individuals with AIS C and D [25,26,27,28]. Additionally, the safety of applying EES for motor function recovery in individuals with SCI has been demonstrated [14,15,17]. However, there are still a limited number of clinical cases and treatment for SCIs of different severities is lacking [29].

Previous studies have indicated that individuals with SCI who regain the ability to walk likely show non-functional gait characteristics, including a slow speed, insufficient endurance, asymmetrical walking, and limited mobility confined to short distances within the house [30,31]. Walking non-functionally is a nonpractical mobility method, with an increased probability of falls or other accidents [32,33,34,35]. However, current traditional rehabilitation training commonly emphasizes compensatory strategies or external devices for non-remediable deficits, which may further distort the walking ability of individuals with SCI [36]. Thus, enhancing the walking function is vital for facilitating the transition into the community.

**Figure 1:**
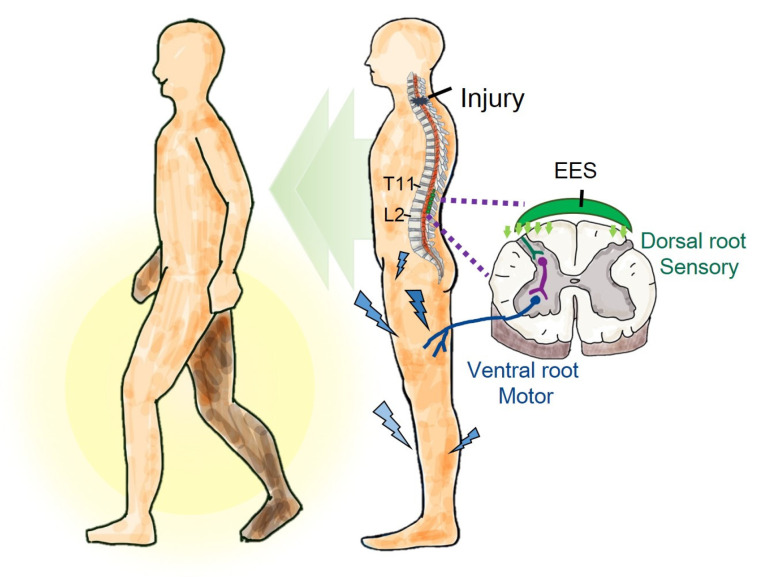
Graphical abstract. In this study, epidural electrical stimulation (EES) substantially increased muscle strength and improved sensory deficits and walking asymmetry. This indicates that EES may improve walking function in individuals with incomplete spinal cord injury.

This study focused on analyzing the effectiveness of EES in improving the muscle strength, sensory recovery, and walking function in individuals with low-severity SCI. Specifically, surface electromyography (sEMG) and gait were assessed and analyzed before surgery, after a short-term EES intervention (8 weeks), and after a long-term EES intervention (36 weeks). The AIS sensory scale could evaluate and confirm the effectiveness of EES in sensory recovery. Additionally, this study used commercially available spinal epidural electrodes instead of a self-designed spinal epidural electrode device previously reported [28]. Accordingly, a further objective of this study was to strategically regulate the electrode configuration and parameters to ensure personalized electrical stimulation parameters during walking. The same approach may help enhance walking function in individuals with incomplete AIS D SCI. Figure 1 shows the graphical abstract.

## 2. Materials and Methods

### 2.1. Inclusion Criteria

Participants with incomplete SCI met the following inclusion criteria: (1) a stable medical condition without cardiopulmonary disease; (2) no musculoskeletal dysfunction and deformities and pressure sores; (3) no clinically significant depression or ongoing drug abuse; (4) no progressive SCI; (5) AIS C or D; (6) functional segmental reflexes below the lesion; (7) spasticity with a grade ≤ 2 on the Modified Ashworth Scale (MAS) in the hip adductors, quadriceps, or gastrocnemius muscle group; (8) no Botox injections in the previous 6 months; (9) an injury duration of ≥1 year; and (10) 20–70 years of age (men or women).

### 2.2. Characteristics of Study Participants

Three participants with cervical-level and chronic SCI were enrolled from 2020 to 2021 in the study approved by the institutional review board of Tzu Chi General Hospital, Hualien, Taiwan (IRB109–082-A) (approved on 1 May 2020), and clinical trial (NCT05433064) (approved on 27 June 2020), with a range in the neurological level from C4 to C5. The mean injury duration was 4.8 years (range of 1.5–10 years), and the mean age at the time of implant was 56.7 ± 9 years (range of 51–67 years) (Table 1). The preoperative bone mineral density (BMD) T-scores of P2 and P3 were 1.9 and −0.6, respectively, while P1 did not undergo examination. In terms of walking ability, participants P1 and P3 walked with a crutch and a walker, respectively; participant P2 could walk independently. All participants walked unsteadily with speeds of <0.44 m/s [37], failing to meet community standards. Furthermore, participant P1 had experienced two falls over the previous six months. The participants underwent 2–3 conventional physiotherapy sessions weekly before recruitment. Spasticity was assessed using the Modified Ashworth Scale (MAS), and the following grades were recorded for each participant: Participant P1’s MAS score was 1+ for the knee flexor and 1 for the ankle dorsiflexor. Participant P2’s MAS score was 1+ for the hip adductor and 1+ for the ankle plantar flexor. Both participants were taking Baclofen, 5 mg BID. Participant P3’s MAS score was 1+ for the ankle plantar flexor, and she was taking Baclofen, 5 mg BID, and Tizanidine, 2 mg BID.

### 2.3. AIS Evaluation

Various physiotherapists independently performed a clinical evaluation following the international standards for the neurological classification of spinal cord injury to check their neurological status using the American Spinal Injury Association (ASIA) impairment scale (AIS). In addition to lower limb motor scores, sensory scores were determined using light touch (LT) and pinprick (PP) tests on the left and right lower limbs below the lesion location. AIS assessments were performed before implantation and after an EES intervention for 8 and 36 weeks. The lower extremity motor (LEM) and sensory scores of EES-stimulated L1–S1 at different times were compared to confirm the clinical efficacy of EES.

### 2.4. Surgical Procedure

All three participants underwent a thoracolumbar laminectomy for the first time for spinal epidural electrode implantation. The final position was reached based on intraoperative mapping to ensure coverage of the L1 to S2 spinal cord segments and to activate lower limb muscles. All participants were allowed to resume daily physical activity on postoperative day 3 and were instructed to wear a back brace to prevent the migration of epidural electrodes [38].

### 2.5. Intraoperative Mapping

In this study, we used an epidural spinal cord stimulation unit (Abbott, Chicago, IL, USA) with a paddle 16-electrode array (Tripole Lead-3219). The stimulation unit was generally implanted between the T11 and L2 vertebral levels, ensuring coverage of the L1 to S2 spinal cord segments to activate the lower limb muscles. Considering individual differences, we used the following stimulation parameters (a 2 Hz frequency and a pulse width of 200 µs) during intraoperative mapping, along with needle electromyography (EMG), to determine the final placement. The key muscles monitored were the iliopsoas, rectus femoris (RF), tibialis anterior (TA), semimembranosus, and soleus. In the intraoperative mapping procedure, we ensured that the iliopsoas of the proximal segment and the soleus of the distal segment had the corresponding electrode configuration to be activated [14,21,28,38].

### 2.6. Spinal Segmental Mapping

**Figure 2:**
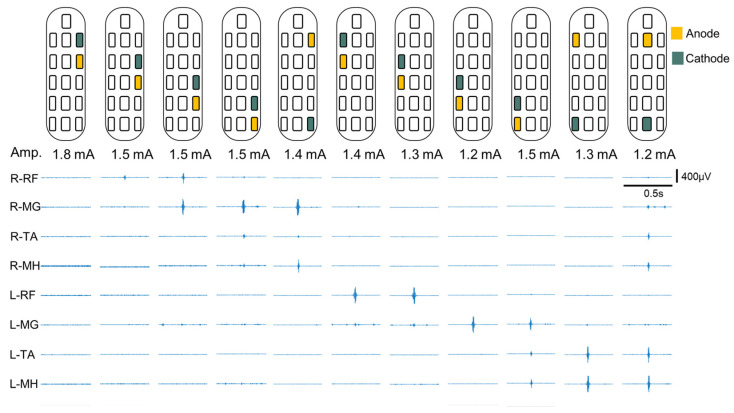
EMG activity of spinal segmental mapping. sEMG response amplitude of muscle groups quantified through peak-to-peak measurements; sEMG responses were evoked with minimum EES amplitude. These results could be used to confirm the muscle group corresponding to the electrode configuration and obtain spinal segmental mapping information. The lower limb muscles stimulated include the iliopsoas, rectus femoris (RF), medial hamstring (MH), tibialis anterior (TA), and medial gastrocnemius (MG). Abbreviations: Amp., amplitude.

Spinal segmental mapping was aimed at determining which electrodes evoked specific muscular contractions and the corresponding effects on the lower limbs [12,14,16,28,39]. During postoperative spinal segmental mapping, a frequency of 2 Hz and a pulse width of 200 μs were used, and the following muscles were observed using sEMG: the RF, TA, medial hamstring (MH), and medial gastrocnemius (MG). Figure 2 shows that different electrode configurations evoke EMG responses in various muscle groups. Although larger amplitudes may produce more pronounced sEMG responses, they can also trigger peripheral muscular contractions. This may lead to false readings of the electrode configuration mapping related to muscle contractions. Therefore, in this study, the sEMG responses evoked by electrode configurations with the minimum amplitude were used for comparison [40]. The peak-to-peak method was used to quantify EMG amplitudes. Each electrode configuration was recorded for 10 s, and the average amplitude of each muscle was calculated. Subsequently, the averaged amplitudes of the muscles stimulated under the same electrode configuration were compared and ranked. The electrode configuration that primarily stimulated the muscle typically exhibited the highest average amplitude. Finally, a personalized electrode configuration could be performed based on the results of spinal segment mapping.

### 2.7. EES Configuration and Intervention

We defined target electrode configurations to assist with walking. A moderate neurological impairment is not a full-blown motor or sensory impairment. Accordingly, we considered that the EES strategy should preserve the original configuration and be primarily utilized to enhance the strength of the weak muscles. Physiotherapists and clinicians determined the weak muscle based on a preoperative AIS LEM score ≤ 3; then, spinal segmental mapping was used to set the initial electrode configurations. Subsequently, physiotherapists and clinicians could modify electrode configurations based on the participants’ gait patterns and real-time sEMG. After setting the appropriate electrode configuration, we tested 5–60 Hz frequencies [41]; the appropriate frequency depended on the gait patterns observed by the physiotherapists as well as the sensations in participants without significant paresthesia or sensory complaints [26,27]. The amplitude was higher than the sensory threshold (sensation of paresthesia or vibration) but below the motor threshold, which produces tonic muscle or motor contraction [26,27]. Furthermore, we set up an amplitude range (+/−1.0 mA) that allowed participants to adjust the stimulation intensity to compensate for impedance changes at their discretion. We invested two weeks in determining personalized configuration parameters; simultaneously, with EES assistance, physiotherapists corrected the participants’ gait patterns to reduce compensatory movements, increasing the opportunity to use their weak muscles during walking. As long-term EES may lead to paresthesia [42], we set a cycle of 20 min of stimulation and 20 min of rest in their daily lives. Participants were instructed to use the stimulation cycle for ≥4 h daily. Moreover, the participants maintained their previous rehabilitation exercises during the intervention period.

### 2.8. Gait Evaluation

Participants underwent three trials of a 10-m walk test (10 MWT) preoperatively and at weeks 8 and 36 of the EES intervention [31,37]. Participants P1 and P2 did not use assistive devices during the gait test, whereas P3 needed to use a walker while testing. The GAITRite® system was used to gather gait parameters, including the walk speed (m/s), cadence (steps/min), and step length. The left and right step lengths were used to calculate the symmetry index (SI) [43] to evaluate the walking symmetry as follows:

(1) $$\mathrm{SI}=\;\frac{\mathrm{SLs}-\mathrm{SLw}}{0.5\times \left(\mathrm{SLs}+\mathrm{SLw}\right)}\times 100\%$$

where SLs is the longer step length and SLw is the shorter step length. A small SI represents better bilateral step symmetry, and SI = 0 indicates perfect symmetry [44,45].

### 2.9. Electromyography Measurements for Walking

Muscle activity during walking was recorded simultaneously using eight surface electrodes at a sampling rate of 2000 Hz (Delsys Trigno Avanti, Delsys Inc., Boston, MA, USA); a custom-written MATLAB script (MATLAB 2019 Mathworks, Inc., Natick, MA, USA) was used for analysis. sEMG recordings included the RF, medial hamstring (MH), TA, and medial gastrocnemius (MG) [19,22]. A bandpass filter with a frequency range of 10 to 450 Hz and a 4th-order Butterworth filter were used for preprocessing. The root mean square of the rectified EMG signal within a 500 ms window size was used to generate normalized EMG envelopes. The RMS of the step was identified using a double threshold and then the sum of the RMS values under the step region; subsequently, time was normalized based on the RMS detection duration [46,47]. For each trial, 10 steps were selected to compute the mean EMG activity and its standard deviation for each muscle and used for the comparison of pre- and postoperative measurements.

### 2.10. Statistical Analysis

The Kruskal–Wallis test was used for multiple comparisons of EMG activity pre-operation and at 8 and 36 weeks after the EES intervention for participants P2 and P3, with a *p* < 0.05 considered to indicate a statistically significant difference. Subsequently, Dunn’s test was applied for post hoc analysis, with the statistical significance adjusted for multiple comparisons using the Bonferroni method. As a result, the findings were considered significant at *p* < 0.016. Because the preoperative sEMG data of participant P1 got lost due to technical issues, the Wilcoxon rank sum test was used to compare the different times after the EES intervention, with significance set at *p* < 0.05 In addition, generalized estimating equations (GEEs) are an effective method for handling correlations in repeated measurement data, facilitating the exploration of changing trends over time. We employed GEEs to facilitate the comparison of gait parameter changes before and after 8 and 36 weeks of EES intervention. The significance level was set at 0.05.

## 3. Results

In this study, we simultaneously analyzed gait parameters and EMG activity during walking. Additionally, we compared the outcomes before implantation to those during EES to evaluate the effectiveness of EES in participants with AIS D.

### 3.1. EES Implantation and Configuration

**Figure 3:**
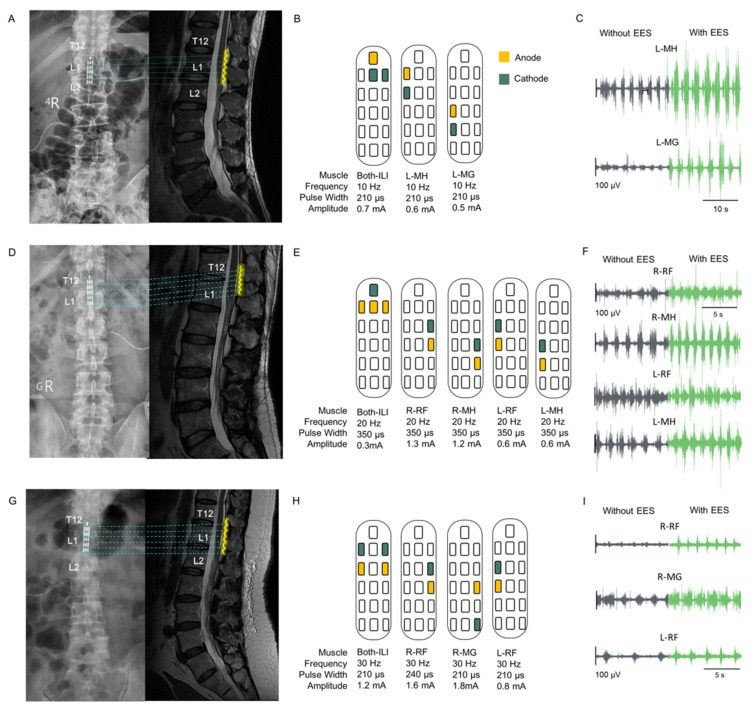
Implant location, configuration, and result of EES muscle stimulation. (**A**,**D**,**G**): Postoperative fluoroscopy and MR images in the sagittal view of the thoracic–lumbar spine, showing the location of the paddle electrode; yellow wavy lines indicate the paddle electrode. The implant locations of the three participants covered the region from thoracic spine 12 (T12) to lumbar spine 2 (L2). (**B**,**E**,**H**): The configurations and parameters of EES for evoking contractions in specific muscles of the lower limbs are displayed. Yellow and green squares indicate the anode and cathode, respectively. The lower limb muscles stimulated include the iliopsoas, rectus femoris (RF), medial hamstring (MH), tibialis anterior (TA), and medial gastrocnemius (MG). (**C**,**F**,**I**): EMG signals illustrating muscle activities in specific muscles without and with EES. The interval between EMG recordings without and with EES was 1 min. Gray and green colors represent EMG signals without and with EES, respectively.

Participant P1 was implanted with a paddle electrode at the L1–L2 vertebral level, whereas participants P2 and P3 were implanted at the T12–L1 vertebral level. Figure 3A,D,G show MR images in the sagittal view from the thoracic–lumbar (T–L) spine level and the corresponding fluoroscopy images indicating the location of the paddle electrode with respect to the bone structure.

The electrode configuration was based on physiotherapists’ suggestions and EMG activity, as shown in Figure 3B,E,H. In addition, Figure 3C,F,I show sEMG waveforms without and with EES when the parameters were configured postoperatively.

### 3.2. AIS Evaluation

An AIS evaluation was conducted preoperatively and at week 8 and 36 of EES. Since EES targets lower limb stimulation, we analyzed the LEM and sensory scores from L2 to S1; the complete AIS assessment with detailed scores is shown in Table 2.

**Figure 4:**
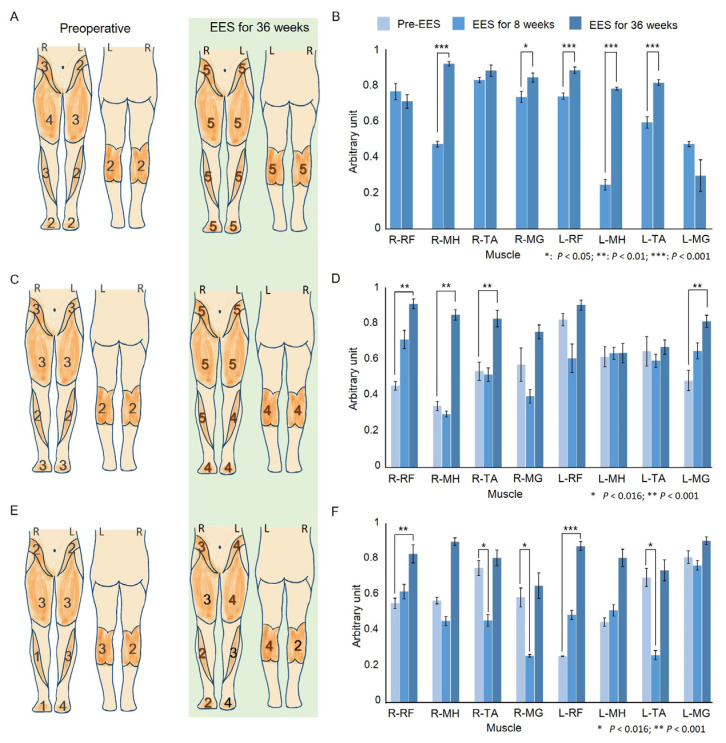
Comparative analysis of clinical assessments and muscle activity pre- and post-EES intervention. (**A**,**C**,**E**): ASIA impairment scale scores for the lower extremities for each muscle. The diagrams on the left and right show the patient’s score on the AIS assessment pre- and post-EES intervention for 36 weeks. (**B**,**D**,**F**): The muscle activity corresponded to the mean of time- and amplitude-normalized RMS envelopes for 10 steps preoperatively and at week 8 and week 36 of treatment with EES. The same EES parameters were used at all assessment times. A device error resulted in missing preoperative EMG data for participant P1; thus, the Wilcoxon rank sum test was used to compare only the two treatment time points. * *p* < 0.05; ** *p* < 0.01; *** *p* < 0.001. The EMG activity of P2 and P3 analyzed using the Kruskal–Wallis test for multiple comparisons, followed by a post hoc Dunn’s test and Bonferroni adjustment. Asterisks indicate significant differences: * *p* < 0.016; ** *p* < 0.001.

The LEM scores of the three participants continued to increase from week 8 to 36 of the EES intervention (Figure 4B). Participant P1 exhibited full scores for both feet at week 36, and P2’s score increased by 12 and 19 points, respectively, at week 8 and week 36 (Figure 4A,C). The overall score of participant P3 continued to increase during EES (preoperative = 24; week 8 and week 36 = 30 and 31, respectively), although with a difference between the left and right scores (left was greater). Notably, regarding the sensory score for L1–S1 according to the AIS, the P1 participant maintained the full score at the preoperative assessment; the PP score of the P2 participant improved by 3 points on both sides at week 8 of the EES intervention and improved to the full score by week 36. Participant P3’s preoperative LT and PP scores were 50% of the full score. The LT score was highest at week 8 of EES intervention, and the PP score of the right side improved by eight points. By week 36, the LT and PP scores of both feet were the highest. Finally, the sensory scores of P2 and P3 reached the full score regardless of the LT or PP, indicating a sensory improvement for L1–S1.

### 3.3. EMG Activity

Due to an unforeseen technical issue during the preoperative sEMG data collection process, the sEMG data of P1 could not be analyzed. Consequently, for P1, we employed a Wilcoxon rank sum test for comparison between weeks 8 and 36 after EES (Figure 4B). Notable increases in performance were observed in the right MH (*p* < 0.001) and MG (*p* = 0.02) and in the left RF (*p* < 0.001), MH (*p* < 0.001), and TA (*p* < 0.001). Further, a significant improvement in the EMG activity was observed in all right leg muscles and the left MG in participant P2 (right RF: *p* < 0.001; MH: *p* < 0.001; TA: *p* = 0.002; and left MG: *p* = 0.001). The post hoc Dunn’s test revealed significantly higher performance in the right RF (*p* < 0.001), MH (*p* < 0.001), TA (*p* = 0.009), and left MG (*p* = 0.001) between preoperative and postoperative measurements. However, at week 8, there were no differences in the EMG activity compared to the preoperative value; week 8 and week 36 showed significant differences in the right MH (*p* < 0.001), TA (*p* = 0.004), and MG (*p* = 0.006) as shown in Figure 4D.

For participant P3, we also detected a significant increase in the EMG activity for all lower limb muscles (right RF: *p* = 0.004; MH: *p* < 0.001; TA: *p* = 0.001; MG: *p* < 0.001; left RF: *p* < 0.001; MH: *p* < 0.001; TA: *p* < 0.001; MG: *p* = 0.017). The post hoc Dunn’s test showed significantly lower EMG activity at week 8 than preoperatively in the right TA (*p* = 0.01), MG (*p* = 0.005), and left TA (*p* = 0.003). However, significant improvements were observed between weeks 8 and 36 in the right MH (*p* < 0.001), TA (*p* = 0.001), and MG (*p* = 0.001) and left MH (*p* = 0.013), TA (*p* = 0.001), and MG (*p* = 0.014). Moreover, sEMG revealed a significant improvement in the right RF (*p* = 0.003), left RF (*p* < 0.001), and MH (*p* < 0.001) between the preoperative measurements and week 36 (Figure 4F).

### 3.4. Gait Performance

In this study, gait parameters showed a non-significant increase in speed and cadence from the preoperative period to 8 to 36 weeks postoperatively. In contrast, the results of the SI demonstrated an increase in the SI in week 8, which was followed by a crucial decrease in week 36. The results of the GEE indicated a significant improvement in the SI (*p* = 0.003) for under 36 weeks. The data summarized indicated an improvement in walking asymmetry. Supplementary Table S1 presents the results of the gait test.

**Figure 5:**
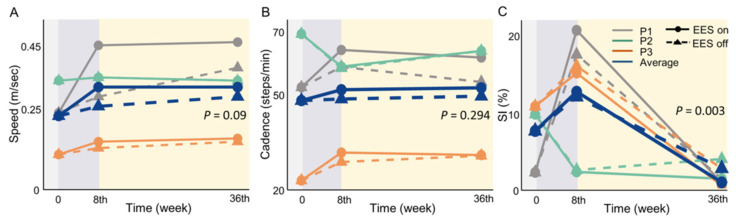
Improvement trend in gait parameters at week 8 and week 36 of EES treatment. Gray line: participant P1; green line: participant P2; orange line: participant P3; blue line: average of all three participants; solid line: with EES; dashed line: without EES. *p* -value based on generalized estimation equations (GEEs). (**A**): increased speed in P1; an advance in P2 and P3 is not apparent (*p* = 0.09). (**B**): cadence outcomes showing a flat trend following EES (*p* = 0.294). (**C**): despite an increase in the symmetry index (SI) in the 8th week, indicating a greater degree of walking asymmetry than preoperatively, all participants showed an improvement in walking symmetry at 36 weeks (*p* = 0.003), with a level of symmetry superior to that observed preoperatively.

The speed and cadence of P1 showed an upward trend from week 8 to week 36 of EES intervention, demonstrating a greater improvement with EES than without EES (Figure 5A,B). The percentage improvement in speed was 92.5% with EES and 57.7% without, while the improvement in cadence was 18.1% with EES and 3.2% without. The SI value of P1 increased at week 8, regardless of stimulation, indicating that the gait exhibited asymmetrical characteristics. Nevertheless, at week 36, the SI value decreased to 0.62% with EE.; the improvement compared to preoperative conditions was 68.7%, indicating an improvement in walking symmetry, and 11.2% without EES.

Participant P2 displayed no improvement in speed and cadence with EES. Nonetheless, there was an obvious improvement in walking symmetry based on the SI. The walking symmetry improved by 88% with EES at week 36. In Participant P3, the speed and cadence were advanced slightly, especially when EES was used, as shown in the Supplementary Table S1. Although the SI increased in week 8, it decreased with or without EES in week 36; both of the improvements compared to preoperative conditions were 77% approximately, which indicated better walking symmetry after the EES intervention.

In this study, we simultaneously analyzed gait parameters and EMG activity during walking. Additionally, we compared the various parameters before and after EES treatment to evaluate the effectiveness of EES in participants with AIS D.

## 4. Discussion

The present study showed an effective and sustained walk function improvement in participants with incomplete SCI and AIS D during 36 weeks of EES. The present findings suggest an improvement in most gait parameters, particularly walking symmetry, in all participants. Additionally, the AIS grade according to the lower extremity sensorimotor scale exhibited a notable advancement, and most muscle activity, when receiving EES, exhibited a significant increase.

### 4.1. EES Implantation

The position of the implanted electrode paddles is crucial for EES therapy [28,48]. Previous studies have demonstrated a correlation between the broader coverage of the lumbosacral enlargement by the electrode paddle and the postoperative recovery of motion and the number of voluntary joint movements [48]. This study determined the position of the electrode paddles through intraoperative mapping. Postoperative images showed that the electrode paddles covered the maximal lumbosacral region for all participants, indirectly confirming the appropriateness of the electrode paddle implantation and intraoperative mapping procedures.

### 4.2. Walk Function Improvement

Improving walking is important to help individuals with SCI return to the community and improve their quality of life. Muscle strength and balance are the most critical factors affecting walking function [49,50]. This study applied EES exclusively to target weak muscles involved in SCI participants, diverging from the spatiotemporal EES used in previous studies [18,28]. Furthermore, EES was turned on continuously during daily activities; participants reported no discomfort from EES. In our study, EMG activity during walking caused a significant enhancement in weak muscles after 36 weeks of EES intervention. Concurrently, the walking performance slightly improved in terms of the speed and cadence after EES. More importantly, previous studies have shown that walking symmetry is a crucial parameter for evaluating walking function [35]. The excellent improvement in walking symmetry reinforces that EES positively influences walking function. The results of this study are consistent with previous research, which demonstrated that EES enhances muscle strength and improves sensory deficits, while suggesting that EES may be beneficial for walking [8,13,29]. Additionally, advances in walking function may be associated with reduced spasticity, muscle atrophy, or metabolic changes in muscle fibers. However, walking asymmetry increased at week 8 with an increasing step length on both sides. This might result from the preoperative shorter step length becoming the longer step length, as illustrated in the Supplemental Figure S1. At week 36, the SI reversed, showing a significant improvement in gait stability. Accordingly, EES may require an adaptation period to facilitate improvements in walking function.

### 4.3. AIS Assessment Improvement

The above muscle activity and gait assessments showed an improved walking ability. In terms of AIS, a previous study showed that increased LEMS in individuals with incomplete motor injuries are often associated with an improved walking ability [49,51]. In addition, balance is critical to walking function recovery in patients with incomplete SCI, where balance impairment is significantly associated with sensory impairment [50]. Therefore, the increase in sensory scores observed here indicated an improvement in balance and an increase in LEM scores, both important improvement measures of walking ability.

Previous studies have identified muscle weakness and impaired balance as primary biological factors contributing to falls in individuals with SCI [30,33]. Additionally, walking asymmetry is a crucial factor increasing the fall risk [33,34,35]. A previous study indicated that fall individuals with SCI showed a significantly lower AIS sensory score of the lower extremities than non-fall individuals [52]. Thus, EES may mitigate fall risk through the amelioration of sensory deficits in the lower limbs. For example, participant P1 had a history of multiple falls before EES. However, after the intervention, the participant no longer suffered falls and exhibited an enhanced ambulatory capacity.

### 4.4. Limitations

This study has some limitations. First, the number of participants included in the study was small, and missing EMG data were noted in participant P1; the walking function evaluation and fall investigation were not comprehensive enough, as they lacked endurance tests, walking assessment scales, fall event statistics analysis, etc. Additionally, the study lacked the use of a paired pulse test using transcranial magnetic stimulation to evaluate the spinal cord function and cortical plasticity and to confirm the nature of evoked signals. Finally, although our proposed EES method may improve walking function, the follow-up period may not be sufficiently long to allow for a more definite conclusion. Future research should include a long-term follow-up (>36 weeks) and a larger group of participants with incomplete SCI to further validate the benefits of EES for individuals with SCI and unstable gait. Additionally, more work is needed to enhance the effectiveness of electrode configurations and the selection of stimulation parameters.

## 5. Conclusions

This study suggests that EES ameliorates sensory impairment and enhances muscular activity as well as muscular strength, which may lead to improved walking symmetry. Furthermore, our findings imply that EES contributes to improved walking function in subjects with incomplete SCI.

## Figures and Tables

**Table 1:** Participant characteristics.

Participant	Age at Implant (yr.)	Sex	Duration of Injury (yr.)	AIS Grade	Neurological Level of Injury	BMD T-Score
P1	52	Male	1.5	D	C4	No test
P2	51	Male	10	D	C5	1.9
P3	67	Female	3	D	C4	−0.6

Abbreviations: AIS, American Spinal Injury Association impairment scale from the international standards for the neurological classification of spinal cord injury; yr.: years; BMD, bone mineral density.

**Table 2:** Participants’ neurological status.

Participant	P1			P2			P3		
Time point	Pre	8th	36th	Pre	8th	36th	Pre	8th	36th
AIS	D	D	D	D	D	D	D	D	D
Neurological levels of injury	C5	C5	C5	C4	C5	L4	C5	C5	C5
UEMs	R | L	R | L	R | L	R | L	R | L	R | L	R | L	R | L	R | L
C5	5 | 4	5 | 4	5 | 4	4 | 4	3 | 4	5 | 5	4 | 4	4 | 4	4 | 4
C6	5 | 4	5 | 4	5 | 4	4 | 4	4 | 3	5 | 5	2 | 3	2 | 3	3 | 3
C7	4 | 4	4 | 4	4 | 4	4 | 4	4 | 3	5 | 5	3 | 3	3 | 3	3 | 4
C8	5 | 4	5 | 4	5 | 4	4 | 4	4 | 4	5 | 5	3 | 3	3 | 4	3 | 3
T1	4 | 4	4 | 4	4 | 4	4 | 4	4 | 3	5 | 5	2 | 4	2 | 4	3 | 3
Total (max. 50)	23 | 20 43	23 | 20 43	23 | 20 43	20 | 20 40	19 | 17 36	25 | 25 50	14 | 17 31	14 | 18 32	16 | 17 33
LEMs	R | L	R | L	R | L	R | L	R | L	R | L	R | L	R | L	R | L
L2	3 | 2	4 | 4	5 | 5	3 | 3	4 | 4	5 | 5	2 | 2	2 | 2	3 | 4
L3	4 | 3	4 | 3	5 | 5	3 | 3	4 | 4	5 | 5	3 | 3	3 | 5	3 | 4
L4	3 | 2	4 | 3	5 | 5	2 | 2	4 | 4	5 | 4	1 | 3	2 | 4	2 | 3
L5	2 | 2	4 | 3	5 | 5	3 | 3	3 | 4	4 | 4	1 | 4	2 | 5	2 | 4
S1	2 | 2	4 | 3	5 | 5	2 | 2	3 | 4	4 | 4	2 | 3	1 | 4	2 | 4
Total (max. 50)	14 | 11 25	20 | 16 36	25 | 25 50	13 | 13 26	18 | 20 38	23 | 22 45	9 | 15 24	10 | 20 30	12 |19 31
Light touch sensory scores	R | L 14 | 14	R | L 14 | 14	R | L 14 | 14	R | L 14 | 14	R | L 14 | 14	R | L 14 | 14	R | L 7 | 7	R | L 14 | 14	R | L 14 | 14
L1–S2									
Pinprick touch sensory scores	R | L 14 | 14	R | L 14 | 14	R | L 14 | 14	R | L 7 | 7	R | L 10 | 10	R | L 14 | 14	R | L 7 | 7	R | L 13 | 7	R | L 14 | 14
L1–S2									

Abbreviations: AIS, American Spinal Injury Association impairment scale from the international standards for the neurological classification of spinal cord injury; C, cervical; T, thoracic spine; L, lumbar spine; S, sacral; Pre, preoperative; 8th, week 8; 36th, week 36; R, right; L, left; UEMs, upper extremity motor scores; LEMs, lower extremity motor scores.

## Data Availability

Data will be available upon contacting the author, Y.C., and upon obtaining the necessary approval from the Ethical and Research Committee of Tzu Chi General Hospital, Hualien, Taiwan.

## Supplementary Materials

The following supporting information can be downloaded at https://www.mdpi.com/article/10.3390/biomedicines13010155/s1: Figure S1: Change in step length from preoperative measurements to 36 weeks of EES intervention. Table S1: The results of the gait test.

## Abbreviations

The following abbreviations are used in this manuscript.

AIS	Association impairment scale
ASIA	American Spinal Injury Association
BMD	Bone mineral density
EES	Epidural electrical stimulation
GEE	Generalized estimating equation
LEM	Lower extremity motor
LT	Light touch
MAS	Modified Ashworth Scale
MG	Medial gastrocnemius
MH	Medial hamstring
RF	Rectus femoris
SCI	Spinal cord injury
SI	Symmetry index
TA	Tibialis anterior
