# Supplementary material for: Role of Epidural Electrode Stimulation in Three Patients with Incomplete AIS D Spinal Cord Injury
Source: Biomedicines. 2025 Jan 10;13(1):155. doi: 10.3390/biomedicines13010155 (PMC11762847; doi:10.3390/biomedicines13010155)
Supplement: Supplementary file 1 [file biomedicines-13-00155-s001.zip › biomedicines-3342667-supplementary.pdf]

Supplementary Materials

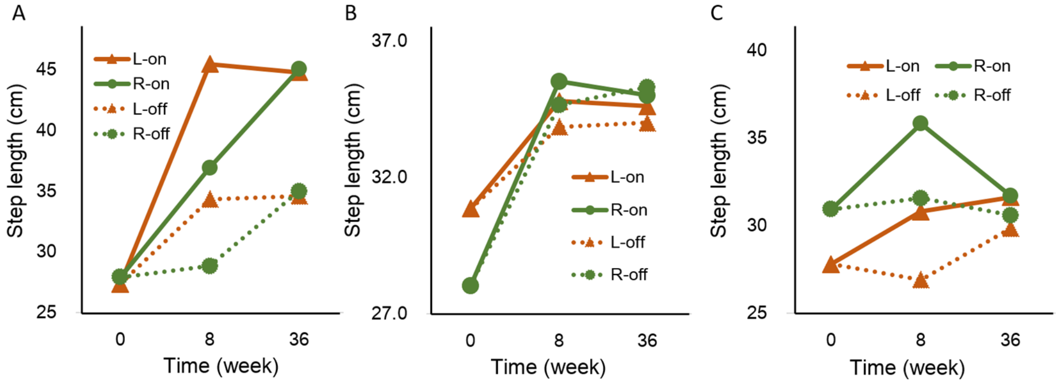

Figure S1. The change of step length from preoperative to 36 weeks of EES intervention. Green line, right side; orange line, left side; solid line: with EES; dish line: without EES. (**A**). Participant P1's step length shows an obvious increase at week 8 and the shorter step reversals to the longer one. In addition, the walking asymmetry increased more than preoperative. In week 36, the shorter length of week 8 increased and was almost equal to the longer step; the state indicated that the step length not only increased but also improved walking symmetry under EES intervention. (**B**). A similar result was observed in participant P2. The only difference is that P2's walking symmetry improved in week 8. (**C**). The step length of participant P3 was increased in week 8, but the shorter step length did not transform into a longer one, and walking symmetry showed improvement in week 36. Abbreviations: L-on, the left leg with EES; R-on, the right leg with EES; L-off, the left leg without EES; R-on, the right leg without EES

Table S1. Gait test.

Gait parameter		Speed (m/s)	Cadence (steps/min)	SI (%)
Participant	EES	Pre / 8th / 36th†	Pre / 8th / 36th†	Pre / 8th / 36th†
P1	Without	0.24 / 0.29 / 0.38	52.4 / 58.9 / 54.1	2.0 / 17.5 / 11.2
	With	0.45 / 0.46	64.3 / 61.9	20.7 / 0.6
P2	Without	0.34 / 0.35 / 0.34	69.4 / 58.2 / 64.0	9.7 / 2.3 / 5.0
	With	0.35 / 0.34	59.0 / 64.0	2.1 / 4.9
P3	Without	0.11 / 0.13 / 0.15	22.7 / 28.6 / 30.6	10.7 / 16.0 / 2.5
	With	0.15 / 0.16	31.6 / 30.8	15.1 / 2.4
Average	Without	0.23 / 0.32 / 0.32	48.2 / 48.7/ 49.6	7.4 / 11.9 / 6.2
	With	0.26 / 0.29	51.6 / 52.2	12.6 / 2.7
p -value‡		0.098	0.294	0.003

Abbreviations: SI, symmetry index; EES, epidural electrical stimulation. Pre / 8th / 36th† : Time points are preoperative, week 8 and 36 of EES intervention *p* -value‡: It is obtained based on the generalized estimation equations (GEE).
